# Frozen shoulder: exploration of terminology and classification

**DOI:** 10.3389/fresc.2024.1498263

**Published:** 2024-11-25

**Authors:** Fabrizio Brindisino, Elif Turgut, Filip Struyf

**Affiliations:** ^1^ Department of Medicine and Health Science “V. Tiberio”, University of Molise, Campobasso, Italy; ^2^ Sports Physiotherapy Department, Faculty of Physical Therapy and Rehabilitation, Hacettepe University, Ankara, Türkiye; ^3^ Department of Rehabilitation Sciences and Physiotherapy, Faculty of Medicine and Health Sciences, University of Antwerp, Wilrijk, Belgium

**Keywords:** classification, terminology as topic, frozen shoulder, shoulder pathology, naming

## Introduction

Frozen Shoulder (FS) is a condition characterized by inflammation of the glenohumeral joint capsule, leading to fibrosis and resulting in functional disability and reduced quality of life (1, 2). Specific landmarks for diagnosis include a Range of Motion (ROM) restriction of at least 25% in at least two movement planes, with more than 50% limitation in external rotation at the arm by the side compared to the unaffected side. Additionally, the symptoms must be stable for at least one month or worsening (3). This condition is estimated to affect up to 10% of the general population, with a higher incidence in subjects aged 40–60 years (4).

FS has been described using various terms and classifications in the medical literature (5). Historically, broad labels like “humeroscapular periarthritis” were used, reflecting limited understanding of its causes. Terms such as adhesive capsulitis, periarthritis, and shoulder contracture are often used interchangeably, highlighting uncertainties about FS pathophysiology and ongoing debates on whether it should be classified by etiology, severity, or other criteria. Inconsistent terminology and heterogeneous samples can limit research efforts, making it challenging to pool data across studies and compare treatment outcomes with confidence in targeting a specific patient group.

In this opinion paper, we reviewed the scientific literature on FS terminology and classification—highlighting the need for a unified terminology to improve communication among researchers and clinicians. Additionally, we proposed new perspectives on the relationship between concurrent conditions and FS.

## Naming

Over 15 different terms have been used in the literature to identify FS, with “frozen shoulder”, “shoulder stiffness”, and “adhesive capsulitis” being the most common (5).

The term “shoulder stiffness” can be misleading, as it describes a clinical phenotype that may result from various conditions —e.g., osteoarthritis, calcific tendinopathy, muscular contracture. Furthermore, no specific clinical history leads to a “stiff shoulder” nor is there an established threshold to define a shoulder as “stiff”. Therefore, while all cases of FS exhibit shoulder stiffness, not all instances of shoulder stiffness qualify as FS.

The term “adhesive capsulitis” aims to describe the underlying pathological process of this condition, but proves to be inaccurate, as adhesions are not consistently observed (5). Additionally, the term “capsulitis” implies a persistent inflammatory process, which is typically only present at the onset of the disease (3, 6). Furthermore, misleading terminology can be detrimental—as it may promote inappropriate treatments, such as adhesion detachment. Notably, international scientific societies, such as ISAKOS (International Society of Arthroscopy, Knee Surgery, and Orthopaedic Sports Medicine) (7) and ASES (American Shoulder and Elbow Surgeons) (8) have advised against using this term; we therefore recommend discarding it as well.

Some researchers argue that “FS” mainly describes the later stages of the condition but accurately reflects the patient's experience —a shoulder that gradually “freezes”, becomes immobile, and then “thaws”, with partial recovery of motion. By implying a generally favorable prognosis, “FS” may also promote patient compliance and adherence to treatment. Additionally, in a broad sense, frozen tissue can cause pain and allodynia (9), potentially explaining the constant, stabbing pain FS patients experience, especially early on. However, the term does not encompass for the initial inflammatory phase that precedes fibrosis. To date, there is no clear consensus on the ideal terminology to capture the etiopathogenesis, clinical presentation, and patient perspective of this condition. However, we suggest that while “Frozen Shoulder” may not be the most precise term, it remains the most suitable, being widely accepted by patients and supported by scientific societies.

## Classification

The literature further categorizes FS into primary (without an identifiable cause) and secondary (with a hypothesized cause), which may include intra-articular factors —e.g., chondral lesions, labral tears, synovitis, or tendonitis of the rotator cuff or biceps— as well as extra-articular factors —e.g., ipsilateral breast surgery, cervical radiculopathy, chest wall tumors, or fractures of the humeral shaft or clavicle— (7, 8). This classification is based not on the anatomical structures involved but rather on the presence of a plausible or conceivable cause (7, 8).

In the authors’ opinion, the presence of a prior intra-articular or extra-articular condition —as mentioned above— followed by the development of FS does not necessarily indicate a causal relationship. Instead, this should be viewed as a chronological association, with current evidence suggesting a possible connection rather than a *definitive* cause. FS could also occur independently of a coexisting pathology, precede it (10) or serve as an early warning sign of its development (11, 12). To date, it may be more appropriate to refer to such conditions as “weak/strong predisposing factors” rather than *causative* ones.

Although empirical observations suggest a possible association, definitive proof is still needed. We therefore recommend using the term “hypothesized” rather than “related” or “associated”, as these imply an *a priori* certainty of correlation that we do not yet have.

The ISAKOS Upper Extremity Council recommends reserving the term “FS” for “primary shoulder stiffness”, while using “secondary shoulder stiffness” for cases with a hypothesized cause. However, we find this distinction unhelpful, as it reduces inter-rater diagnostic agreement (13), and adds little to diagnosis, management, or prognosis. This terminology could also confuse clinicians, leading them to believe the condition is fundamentally different from FS, despite similar clinical presentations and treatments. To create a unified treatment approach —and avoid a “Babylonian confusion of languages” (14) offering little benefit to FS patients— we recommend using “FS” as the sole label, possibly specifying any hypothetically related pathologies.

Some classifications include an extrinsic/intrinsic subtype for “secondary” FS (8): “intrinsic” referring to concurrent conditions within the glenohumeral joint, such as rotator cuff disorders, biceps tendinitis, or calcific tendonitis (7). In contrast, “extrinsic” subtypes refer to FS developing in subjects with one or more conditions *potentially* linked to FS but located outside the shoulder —e.g., ipsilateral breast surgery, cervical radiculopathy, chest wall tumor, cerebrovascular accident— or local extrinsic issues —including previous humeral shaft fracture, acromioclavicular arthritis, or clavicle fracture (7, 8). In our opinion, this sub-classification is both useful and convincing, as comorbidities are present in 85% of FS patients —with 37.5% having more than three comorbidities (6). This sub-classification serves as a reminder for clinicians to consider concurrent pathologies when treating FS, as these may warrant pharmacological, surgical, or therapeutic interventions (15), particularly when multidisciplinary expertise is required. Additionally, specific precautions are needed when treating FS in patients with certain comorbidities, such as recently stabilized fractures or repaired rotator cuff tendons (16).

Another four-arm sub-classification for “FS with a hypothesized cause” was proposed —comprising *intra-articular, capsular, extra-articular*, and *neurologic causes* (7). However, these subtypes could still align with the abovementioned broader “intrinsic” and “extrinsic” categories, which may be preferable for improving inter-rater agreement in classification. Moreover, other classification systems provided *systemic* sub-categorization of FS when associated with systemic disorders—e.g., diabetes mellitus and hyper/hypothyroidism (8). However, this sub-categorization achieved consensus among only some ASES and Korean surgeons (13) and was omitted from ISAKOS paper —as the systemic/metabolic status was not considered a distinct category *per se*.

Accordingly, we believe that certain conditions —such as metabolic system involvement (17), blood glucose availability (12), dysautonomia (18, 19), low psychological mood (20), altered lipid metabolism (21, 22), and a sedentary lifestyle (6)— may predispose individuals to FS. These conditions contribute to a “low-grade of inflammation status”, forming an underlying environment that may promote the onset and progression of FS and influence its prognosis (6, 18, 23).

Overall, the terminology and classification of FS remain varied, with challenges arising from variations in descriptions and nomenclature, leading to debates on the most suitable label. This lack of consensus extends to distinctions between primary and secondary FS, intrinsic and extrinsic subtypes, and systemic associations—complicating diagnosis and treatment strategies.

## Discussion

**Figure 1:**
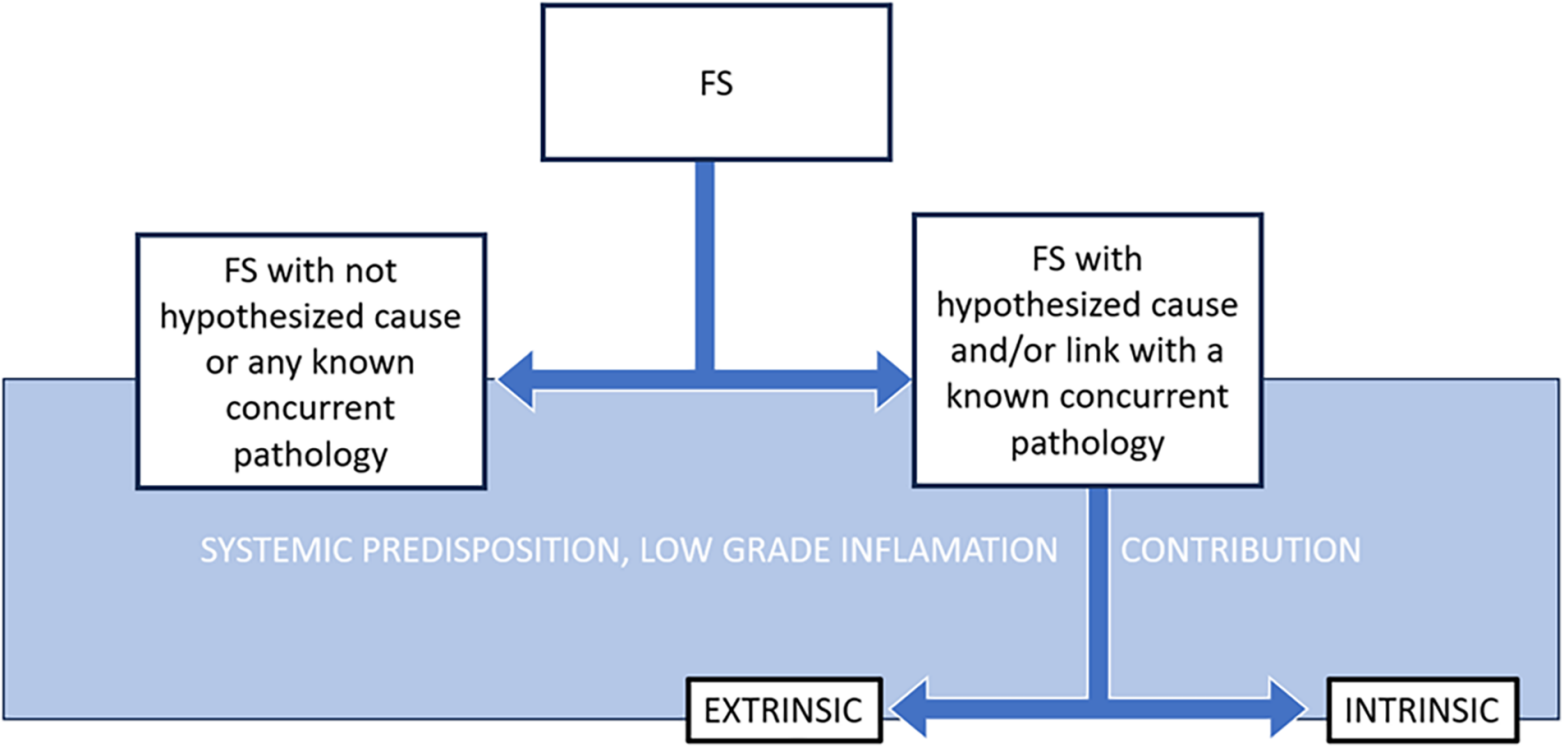
Synopsis of the terminology suggested to (sub-) classify frozen shoulder (FS).

The authors recommend using the term “Frozen Shoulder” as it is accepted by patients and approved from scientific societies. For classification, the authors propose a simplified approach, using “Frozen Shoulder” as the primary label to improve inter-rater agreement in diagnosis and treatment, while specifying any *hypothetically* related pathologies (Figure 1). It is essential to recognize that the “hypothetically related pathology” associated with FS can pose life-threatening risks. This underscores the necessity for fostering multidisciplinary collaboration to ensure optimal patient care. The authors advocate for continuous monitoring of patients’ clinical histories and tracking changes throughout their treatment. It is important to recognize that hypothetically related pathologies may not only precede FS but also occur simultaneously, underscoring the need for thorough monitoring in patient management. Ultimately, it is crucial for the scientific and medical community to continue refining the terminology and classification of FS based on emerging evidence and shared understanding.
